# Activated Microglia Desialylate and Phagocytose Cells via Neuraminidase, Galectin-3, and Mer Tyrosine Kinase

**DOI:** 10.4049/jimmunol.1502532

**Published:** 2017-05-12

**Authors:** Koji Nomura, Anna Vilalta, David H. Allendorf, Tamara C. Hornik, Guy C. Brown

**Affiliations:** Department of Biochemistry, University of Cambridge, Cambridge CB2 1QW, United Kingdom

## Abstract

Activated microglia can phagocytose dying, stressed, or excess neurons and synapses via the phagocytic receptor Mer tyrosine kinase (MerTK). Galectin-3 (Gal-3) can cross-link surface glycoproteins by binding galactose residues that are normally hidden below terminal sialic acid residues. Gal-3 was recently reported to opsonize cells via activating MerTK. We found that LPS-activated BV-2 microglia rapidly released Gal-3, which was blocked by calcineurin inhibitors. Gal-3 bound to MerTK on microglia and to stressed PC12 (neuron-like) cells, and it increased microglial phagocytosis of PC12 cells or primary neurons, which was blocked by inhibition of MerTK. LPS-activated microglia exhibited a sialidase activity that desialylated PC12 cells and could be inhibited by Tamiflu, a neuraminidase (sialidase) inhibitor. Sialidase treatment of PC12 cells enabled Gal-3 to bind and opsonize the live cells for phagocytosis by microglia. LPS-induced microglial phagocytosis of PC12 was prevented by small interfering RNA knockdown of Gal-3 in microglia, lactose inhibition of Gal-3 binding, inhibition of neuraminidase with Tamiflu, or inhibition of MerTK by UNC569. LPS-induced phagocytosis of primary neurons by primary microglia was also blocked by inhibition of MerTK. We conclude that activated microglia release Gal-3 and a neuraminidase that desialylates microglial and PC12 surfaces, enabling Gal-3 binding to PC12 cells and their phagocytosis via MerTK. Thus, Gal-3 acts as an opsonin of desialylated surfaces, and inflammatory loss of neurons or synapses may potentially be blocked by inhibiting neuraminidases, Gal-3, or MerTK.

## Introduction

Phagocytosis is the cellular engulfment of large extracellular particles, including other cells or parts of cells, such as synapses. Cells are phagocytosed by competent phagocytes (cells specialized in phagocytosis) if they possess “eat-me” signals on their surface, lose “don’t-eat-me” signals, and/or bind opsonins (1, 2). Opsonins are soluble proteins that, when bound to cells, promote phagocytosis of those cells. Phagocytosis is greatly upregulated during inflammation, in part due to the release of opsonins (3). Phagocytes can phagocytose dead or dying cells, as well as stressed, pathogenic, damaged, or excess cells, and excessive phagocytosis of otherwise viable cells may contribute to pathology (2, 3). The code that determines whether a particular cell (or cell part) is phagocytosed is still poorly understood but is important in physiology and pathology.

Microglia are CNS-resident macrophages and are the main phagocytes in the brain. In the absence of inflammation, “resting” microglia phagocytose excess neurons and synapses and monitor the brain for damage or pathogens (3, 4). When microglia detect inflammatory stimuli they become “activated,” highly phagocytic, and potentially neurotoxic, and they may contribute to ischemic, traumatic, psychiatric, and neurodegenerative diseases (3, 5, 6). Thus, it is important to understand what determines microglial phagocytosis of neurons and neuronal parts.

Mer tyrosine kinase (MerTK) is a phagocytic receptor found on myeloid lineage cells, including microglia (7). MerTK is upregulated on microglia in response to inflammation (8) and is required for phagocytosis of apoptotic cells (9), stressed neurons (8), and synapses (10). MerTK can be activated by the opsonins growth arrest–specific protein 6 or Protein S bound to phosphatidylserine (7). More recently, galectin-3 (Gal-3) was identified as a MerTK ligand and opsonin (11), but this interaction and what controls Gal-3 binding and opsonization of cells are poorly characterized.

Gal-3, also known as Mac-2 or LGALS3, is a protein expressed in macrophages and microglia, and its expression is increased by inflammatory activation (12–15). Gal-3 is found inside and outside the cell, but the mechanism by which it is released is unclear (16). Gal-3 has an N-terminal tail fused to a carbohydrate-recognition domain (17), which preferentially binds to *N*-acetyl-lactosamine (a disaccharide of galactose and *N*-acetyl-glucosamine) in glycoproteins or gangliosides (18).

The sugar chains of animal glycoproteins and glycolipids normally terminate in a sialic acid residue. Sialic acid is the generic term for derivatives of the monosaccharide neuraminic acid, of which the most common is *N*-acetylneuraminic acid. The terminal sialic acid residue can be removed (a process known as desialylation) by sialidases (also known as neuraminidases [Neus]) to expose *N*-acetyl-lactosamine residues (i.e., the potential binding site for Gal-3) (18). In mammals, there are four sialidases/Neus: Neu1 is found predominantly in lysosomes but also on the cell surface, Neu2 is found in the cytosol, Neu3 is found on the plasma membrane, and Neu4 is found in lysosomes (19). Inflammatory activation of macrophages causes translocation of Neu1 onto the cell surface, where it can desialylate glycoproteins and glycolipids (20, 21). Neu1 is inhibited by Tamiflu, a drug developed to inhibit viral Neus (20). It is not known whether Neu1 is expressed by, or has any role in, microglia.

Apoptotic cells possess a sialidase activity that desialylates the surface and promotes phagocytosis of the apoptotic cell (22). Sialylation may inhibit phagocytosis of the cell by activating Siglec receptors (receptors for sialic acid residues) on phagocytes that inhibit phagocytosis by phagocytes. For example, desialylation of neurons promotes phagocytosis of neurons and neurites by microglia, apparently by reduced activation of inhibitory Siglec-E receptors (23), as well as by complement factors (24).

In this study, we investigated whether and how desialylation and Gal-3 affect the phagocytosis of one cell by another, using BV-2 and PC12 cells as a model system relevant to microglial phagocytosis of neurons (25). As part of this investigation, we have sought to determine whether inflammation causes desialylation of microglia or neurons and, if so, which Neu is involved; whether desialylation of neurons enables Gal-3 binding; whether Gal-3 binding promotes microglial phagocytosis of the neurons and, if so, whether this is via binding MerTK; and whether blocking Neu1, Gal-3, or MerTK prevents microglial phagocytosis of neurons.

## Materials and Methods

### Materials

All chemicals and reagents were from Sigma-Aldrich (St. Louis, MO), unless otherwise indicated. All cell culture and immunoblotting reagents were from Invitrogen (Paisley, U.K.), unless otherwise indicated. Abs (with dilutions for immunoblotting) were polyclonal anti–Galectin-3 (1:500; Santa Cruz Biotechnology, Santa Cruz, CA), anti-MerTK (1:750; FabGennix, Frisco, TX), and anti–β-actin (Cell Signaling Technology, Danvers, MA).

### Cells and treatments

All experiments were performed in accordance with the U.K. Animals (Scientific Procedures) Act (1986) and approved by the Cambridge University local ethical committee. The murine microglial cell line BV-2 (passage < 30) and the rat pheochromocytoma cell line PC12 were maintained as described (26, 27). Where indicated, PC12 cells were differentiated by 50 ng/ml 7S nerve growth factor. Primary mixed glial cultures were prepared from cortex and neuronal–glial culture (85% neurons) was prepared from cerebella of postnatal day-5–7 Wistar rats, as described (28, 29). Dead neurons and neuronal debris were obtained by scraping cells from live neuronal–glial culture (85% neurons) and then passing cells through a 0.4 × 13-mm needle 10 times. The resulting dead neurons and debris were stained with the succinimidyl ester form of TAMRA, and 30 μg (of protein) was added to microglia for 1 h.

BV-2 cells were stimulated with LPS from *Salmonella enterica* serotype typhimurium (100 ng/ml), recombinant Gal-3 (rGal-3; 200 nM), and Neu from *Vibrio cholera* (0.1 U/ml) for 24 h, unless otherwise indicated. For the Gal-3–binding assay, differentiated PC12 cells were treated with staurosporine (500 nM) for 24 h. Where indicated, cells were also treated with various inhibitors (UNC569 [500 nM], Millipore, Billerica, MA; cyclosporin A [CsA; 100 ng/ml]; FK506 [1 μM], Fujisawa Pharmaceutical, Osaka, Japan), Tamiflu (oseltamivir phosphate, 500 μM, unless otherwise indicated; LKT Laboratories, St. Paul, MN), or Cli-095 (5 μM; InvivoGen) for 30 min prior to stimulation. In all cases, inhibitors were used at concentrations predicted to inhibit their target activity by ∼90%, without appreciable off-target effects.

### Phagocytosis and protein detection

Phagocytosis assays were performed as previously described (28–30). Where indicated, 10,000 events were collected per well, and changes in mean FL3 were analyzed using a flow cytometer (Accuri C6 flow cytometer; BD). Western blot studies were performed using standard procedures (31, 32), and membrane-bound secondary Abs were detected using an Odyssey detection system (LI-COR, Lincoln, NE). Where appropriate, culture media were concentrated to ∼40 times using a 10-kDa cut-off filter (Millipore). MerTK–Gal-3 interaction was assessed by coimmunoprecipitation using 250 μg of cellular protein per sample immunoprecipitated by 2 μg of MerTK Ab and Pierce Protein A/G Magnetic Beads (Thermo Scientific, Waltham, MA). Furthermore, MerTK-immunoprecipitated beads were incubated with 200 nM TAMRA-stained rGal-3 at 37°C for 2 h, and mean FL3 was analyzed with an Accuri C6 flow cytometer. A Quantikine ELISA Kit (R&D Systems, Minneapolis, MN) was used to measure TNF-α concentrations in the culture medium, according to the manufacturer’s instructions, and absorbance was measured using a FLUOstar OPTIMA plate reader at 450 nm (specific wavelength) and 570 nm (nonspecific wavelength).

Gal-3 release from cells was analyzed using Western blotting. After LPS stimulation of BV-2 cells, serum-free culture medium was removed and centrifuged at 1250 rpm at 4°C for 5 min. Supernatant was then concentrated to ∼40 times using a 10-kDa cut-off filter. A constant amount of concentrated supernatant and cell lysate was analyzed by Western blot. Data were normalized to β-actin expression in cells (input).

### RNA interference and Neu activity

RNA interference was performed by transfecting 10 nM Gal-3 small interfering RNA (siRNA) or nontargeting control siRNA (Santa Cruz Biotechnology) to BV-2 cells, using Lipofectamine 2000 (Invitrogen), for 24 h. Cells were analyzed using a fluorescence microscope (DMI6000; Leica) or a confocal microscope (IX81; Olympus). Neu activity was assessed using an Amplex Red Neuraminidase Assay Kit (Life Technologies, Carlsbad, CA) and by measuring FITC-conjugated peanut agglutinin (2 μg/ml) binding to the cells.

### Fluorescence polarization

Galectin-3 (Sigma-Aldrich) was labeled with Alexa Fluor 488 NHS Ester (succinimidyl ester) in PBS at room temperature for 30 min, and free dye was washed with desalting spin columns (Thermo Scientific). Labeling was checked by MALDI mass spectrometry. Fluorescence anisotropy measurements were recorded using a PHERAstar Plus multidetection plate reader (BMG Labtech) equipped with a fluorescence polarization optic module (λex  =   485 nm, λem  =  520 nm) at 25°C. Each data point is the mean of 200 flashes per well. The voltage gain was set by adjusting the target mP values of labeled Gal-3 to that of fluorescein (35 mP). Serial dilutions of MerTK (Sino Biological), modified citrus pectin (ecoNugenics), and TREM2 ectodomain were made in PBS and added together in a 1:1 ratio to a constant concentration of Gal-3 (35 nM; 44 mP). *K*_d_ was estimated, assuming a 1:1 binding, using GraphPad Prism. Purified TREM2 ectodomain was a kind gift from Roger Dodd and Peter St. George-Hyslop (University of Cambridge).

### Statistics

Experimental data are expressed as mean ± SEM. Data were analyzed for statistical significance using a general linear model ANOVA with Tukey post hoc analysis using Minitab software. In all cases, statistical significance was defined as *p* < 0.05.

## Results

### Gal-3

**FIGURE 1.**
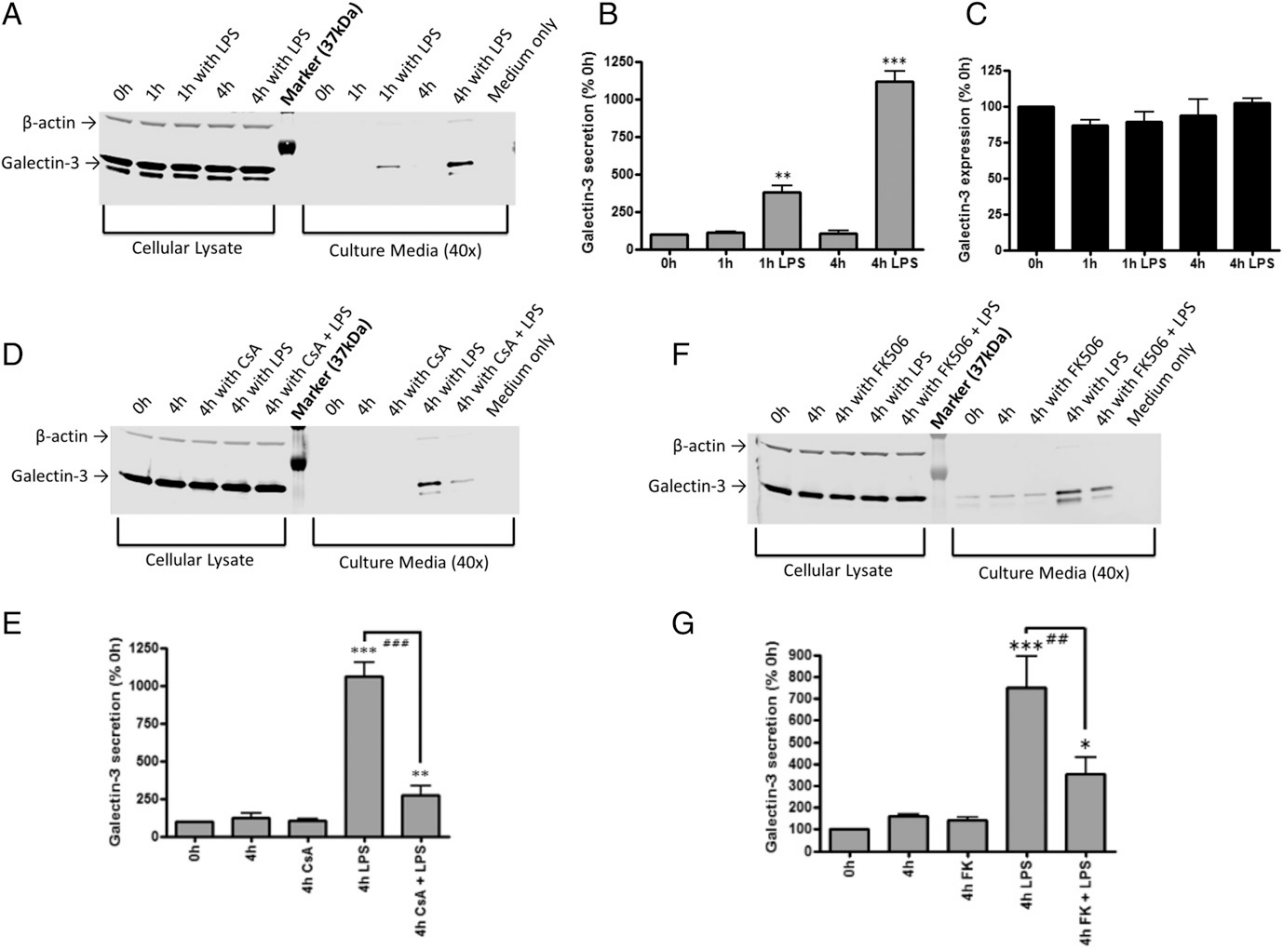
Gal-3 is secreted in response to LPS stimulation. BV-2 cells were exposed to vehicle or 100 ng/ml LPS for 1 or 4 h. (**A**) Representative Western blot showing effect of LPS on Gal-3 expression and secretion. Gal-3 secretion was significantly induced by LPS in a time-dependent manner (1 h, *p* = 0.002 and 4 h, *p* < 0.001) (**B**), but total cellular Gal-3 was unchanged (Tukey post hoc test) (**C**). *n* = 3. Representative Western blot showing the effect of LPS and CsA (**D**) and LPS and FK506 (**F**) on Gal-3 expression and secretion at 4 h (*n* = 3). (**E** and **G**) Quantification of three independent experiments from (D) and (F), respectively. *p < 0.05, **p < 0.01, ***p < 0.001 versus 0 h, Tukey post hoc test. ^###^*p* < 0.01, LPS versus LPS + CsA, ^##^*p* < 0.01, LPS versus LPS + FK506, Tukey post hoc test.

Gal-3 has been reported to mediate macrophage phagocytosis of cells (11) and to be released by microglia (15), so we tested whether Gal-3 mediated microglial phagocytosis. First, we investigated the time course of Gal-3 release from BV-2 microglia in response to the TLR4 activator LPS. We found that LPS (100 ng/ml) induced a strong and rapid release of Gal-3 into the culture media, such that its concentration increased from 100% at the time of LPS addition to 382 ± 41% after 1 h (*p* = 0.002, Tukey post hoc test) and to 1116 ± 41% after 4 h (*p* < 0.001, Tukey post hoc test) (Fig. 1A, 1B). In contrast, LPS had no effect on the protein expression of Gal-3 within microglia (Fig. 1C). Thus, LPS caused Gal-3 release without changing expression over this time course.

Serine phosphorylation of Gal-3 affects its cellular distribution and activity (33), and LPS can activate the serine/threonine phosphatase, calcineurin (34); therefore, we tested whether inhibition of calcineurin would affect Gal-3 release. We found that inhibition of calcineurin by CsA or FK506 significantly attenuated Gal-3 secretion induced by LPS (CsA: *p* < 0.001, FK506: *p* = 0.022) without affecting Gal-3 expression in the cells (Fig. 1D–G).

**FIGURE 2.**
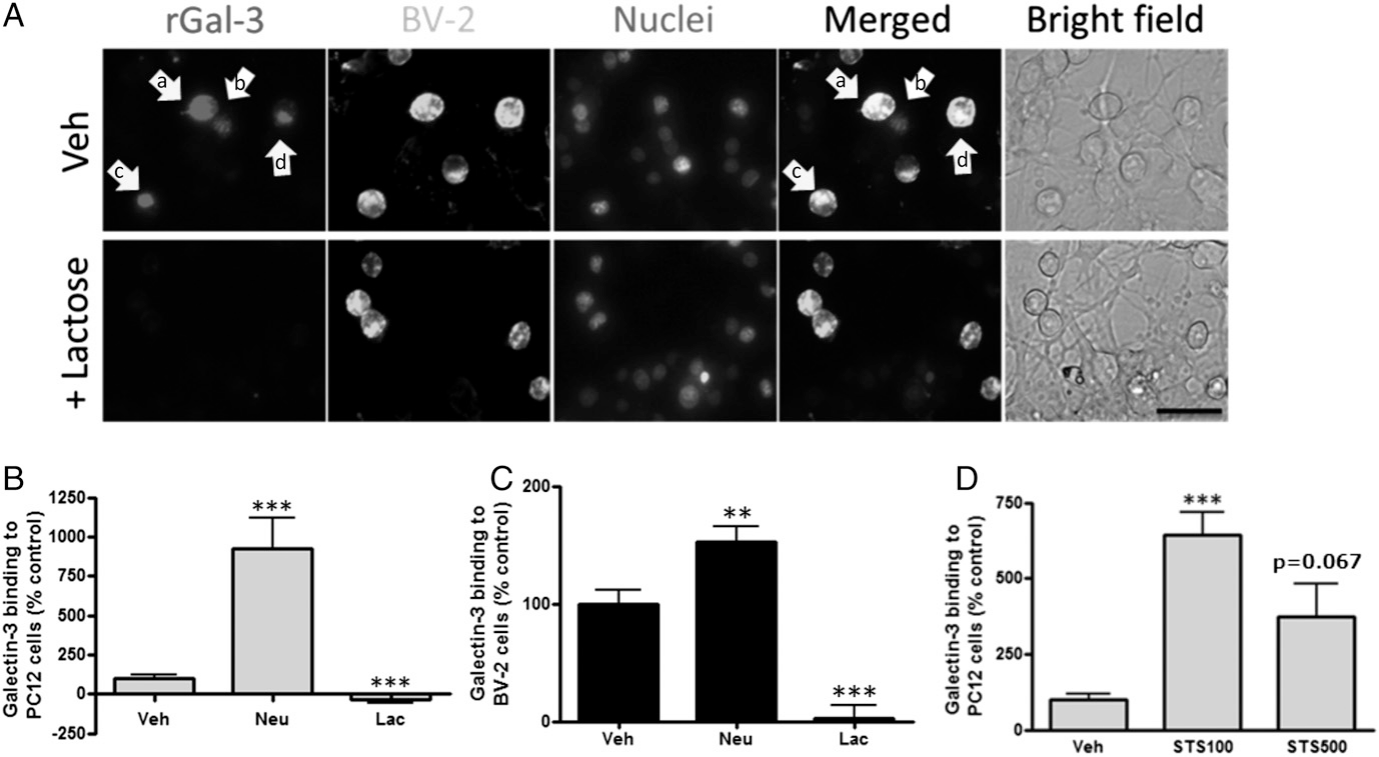
Gal-3 binding to BV-2 and PC12 cells. (**A**) rGal-3 was labeled with TAMRA and added to a coculture of BV-2 and PC12 cells for 2 h. Gal-3 bound to many BV-2 cells (arrow a) but to only some PC12 cells associated with BV-2 cells (arrow b) and what appears to be phagocytosed cells within microglia (arrows c and d). Binding was blocked by lactose (lower panels). Scale bar, 50 μm. PC12 cells (**B**) or BV-2 cells (**C**) were treated with vehicle, Neu (0.1 U/ml), or Neu plus lactose (Lac; 50 mM) for 1 h, followed by 2 h with labeled rGal-3. Flow cytometry was used to quantify Gal-3 binding (*n* = 3). (**D**) PC12 cells were treated with 100 or 500 nM of staurosporine (STS) or vehicle (Veh) for 24 h, TAMRA-labeled rGal-3 was applied to the cells for 2 h, and binding was quantified by flow cytometry. Gal-3 bound more specifically to stressed (+100 nM STS) PC12 cells than to apoptotic PC12 cells (+500 nM STS) (*n* = 3). **p < 0.01, ***p < 0.001 versus vehicle-treated control cells.

To determine whether extracellular Gal-3 binds to cells, rGal-3 was fluorescently labeled and added to a coculture of BV-2 and PC12 cells for 2 h. Fluorescence imaging and flow cytometry indicated that Gal-3 bound to many microglia but only bound to occasional PC12 cells, in particular those closely associated with microglia (Fig. 2A, arrow a) and what appeared to be phagocytosed cells within microglia (Fig. 2A, arrows b, c). Gal-3 binding to PC12 cells and BV-2 cells was completely inhibited by 50 mM lactose (Fig. 2A–C), indicating that Gal-3’s interaction with the cells was entirely mediated by binding to sugar residues.

Whether Gal-3 bound to healthy, stressed, and/or dead cells was tested with PC12 cells treated with the protein kinase inhibitor staurosporine (500 nM). It induced significant cell death of PC12 cells over 24 h, whereas 100 nM staurosporine induced no significant apoptosis or necrosis (Supplemental Fig. 1A). Staurosporine treatment substantially increased Gal-3 binding to PC12 cells, and binding was greater to the “stressed” cells (100 nM staurosporine) than to the “apoptotic” cells (500 nM staurosporine) (Fig. 2D). Thus, Gal-3 binds to stressed but viable cells, as well as to dead cells.

We tested whether Gal-3 binding was regulated by sialylation of the cell surface by adding isolated Neu (0.1 U/ml) to PC12 cells. This resulted in desialylation of the cells, as indicated by the binding of peanut agglutinin (Supplemental Fig. 1B), a lectin that only binds desialylated cells (35). Neu-induced desialylation of the cells also greatly enhanced Gal-3 binding to PC12 cells (Fig. 2B, Supplemental Fig. 2A) and colocalized with peanut agglutinin on the surface of target cells (Supplemental Fig. 2B), suggesting that Gal-3 was binding to desialylated surfaces.

**FIGURE 3.**
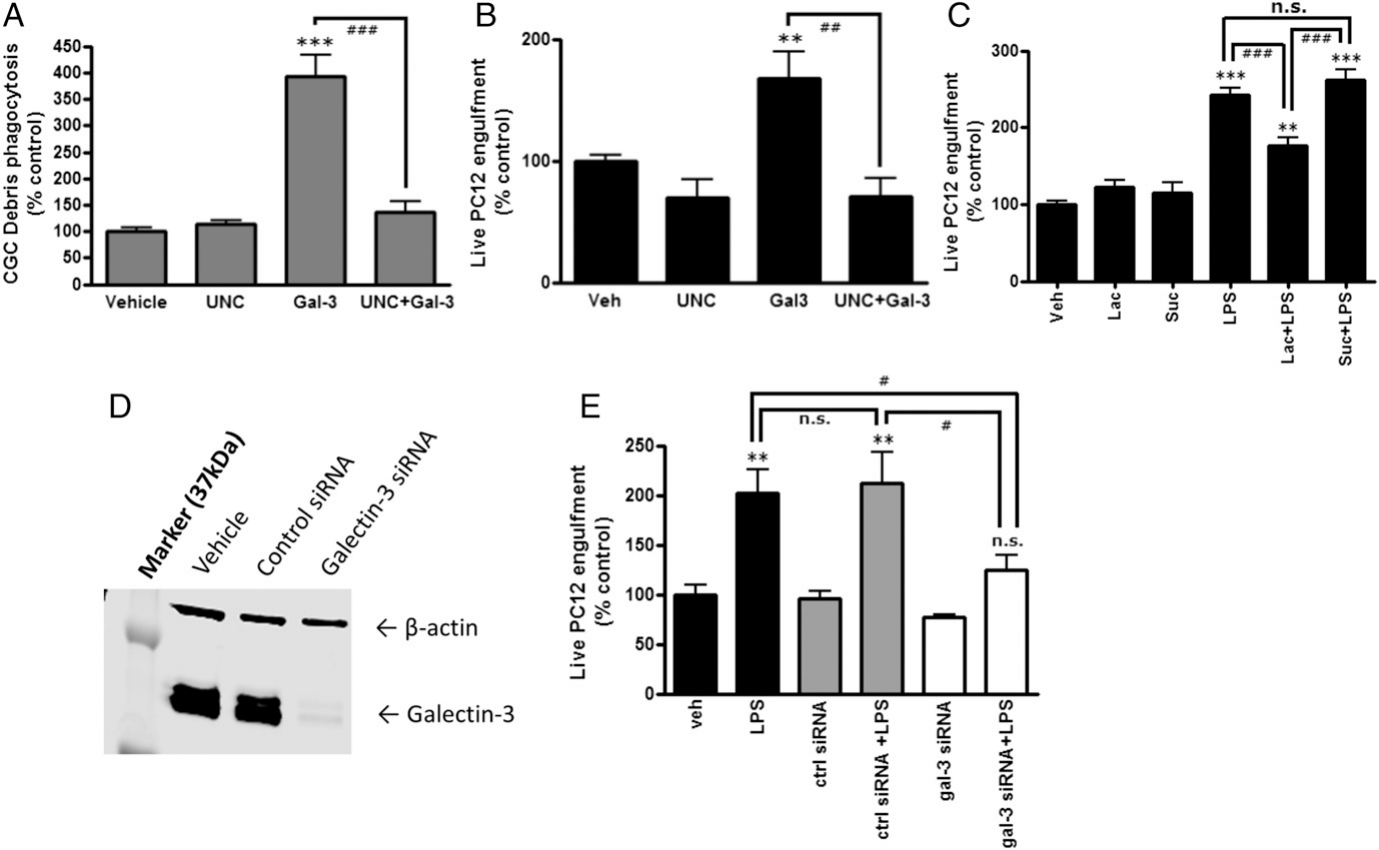
Gal-3 mediates microglial phagocytosis of dead and live neurons. (**A**) BV-2 cells were treated or not with UNC569 (500 nM) and 200 nM Gal-3 for 18 h, neuronal debris was added, and phagocytosis was quantified after 2 h. Absolute phagocytosis of the vehicle control cells: 2.47 ± 0.42% (*n* = 3). ***p < 0.001 versus vehicle control, Tukey post hoc test. ^###^*p* < 0.001 between indicated treatments. (**B**) Alternatively, live PC12 cells were added for 4 h, and BV-2 phagocytosis of PC12 cells was quantified. Absolute phagocytosis of the vehicle control cells: 10.40 ± 1.15% (*n* = 3). **p < 0.01 versus vehicle control. ^##^*p* < 0.01 between indicated treatments. (**C**) LPS-induced phagoptosis of stressed but viable PC12 cells was significantly attenuated by lactose but not sucrose. Absolute phagocytosis of the vehicle control cells: 9.45 ± 1.57% (*n* = 3). **p < 0.01, ***p < 0.001 versus vehicle control. ^###^*p* < 0.001 between the indicated treatments. (**D**) Representative Western blot showing the effect of Gal-3 knockdown. (**E**) LPS-induced phagoptosis of stressed but viable PC12 cells was significantly attenuated by Gal-3 knockdown (*p* = 0.046). Absolute phagocytosis of the vehicle control cells: 9.33 ± 0.83% (*n* = 3). **p < 0.01 versus vehicle control. ^#^*p* < 0.05 between indicated treatments. All *p* values were calculated using the Tukey post hoc test. n.s., not significant.

Gal-3 has been reported to opsonize cells for phagocytosis by macrophages, but it is not known whether this is true for microglial phagocytosis of live or dead neurons. We found that addition of 200 nM rGal-3 substantially increased the phagocytosis of neuronal debris (sheared primary neurons, *p* < 0.001, Tukey post hoc test) and live PC12 cells (*p* < 0.05, Tukey post hoc test) (Fig. 3A, 3B).

Because LPS induced Gal-3 release (Fig. 1A, 1B), and addition of Gal-3 induced microglial phagocytosis of PC12 cells (Fig. 3B), we tested whether LPS induced microglial phagocytosis of PC12 cells and whether this was mediated by Gal-3. We found that LPS did increase phagocytosis of live PC12 cells, and addition of lactose (which blocked Gal-3 binding to PC12 cells, Fig. 2A) inhibited this phagocytosis (*p* < 0.001, Tukey post hoc test) (Fig. 3C). Note that neither LPS nor Gal-3 had any effect on the viability of PC12 cells in the absence of microglia (Supplemental Fig. 1C). Because calcineurin inhibitors decreased LPS-induced Gal-3 release (Fig. 1D–G), we also tested whether they would change LPS-induced phagocytosis of cells; we found that CsA and FK506 inhibited phagocytosis of cells (Supplemental Fig. 3A, 3B). To directly test the role of Gal-3 in LPS-induced phagocytosis, Gal-3 was knocked down by siRNA to 6.5 ± 0.8% of its control protein expression (Fig. 3D). Gal-3 knockdown significantly inhibited LPS-induced phagocytosis of PC12 cells (*p* < 0.05, Tukey post hoc test) (Fig. 3E). Thus, overall, the data indicate that LPS causes microglia to release Gal-3, which binds to stressed or dead neurons, opsonizing them for phagocytosis by microglia.

### MerTK

**FIGURE 4.**
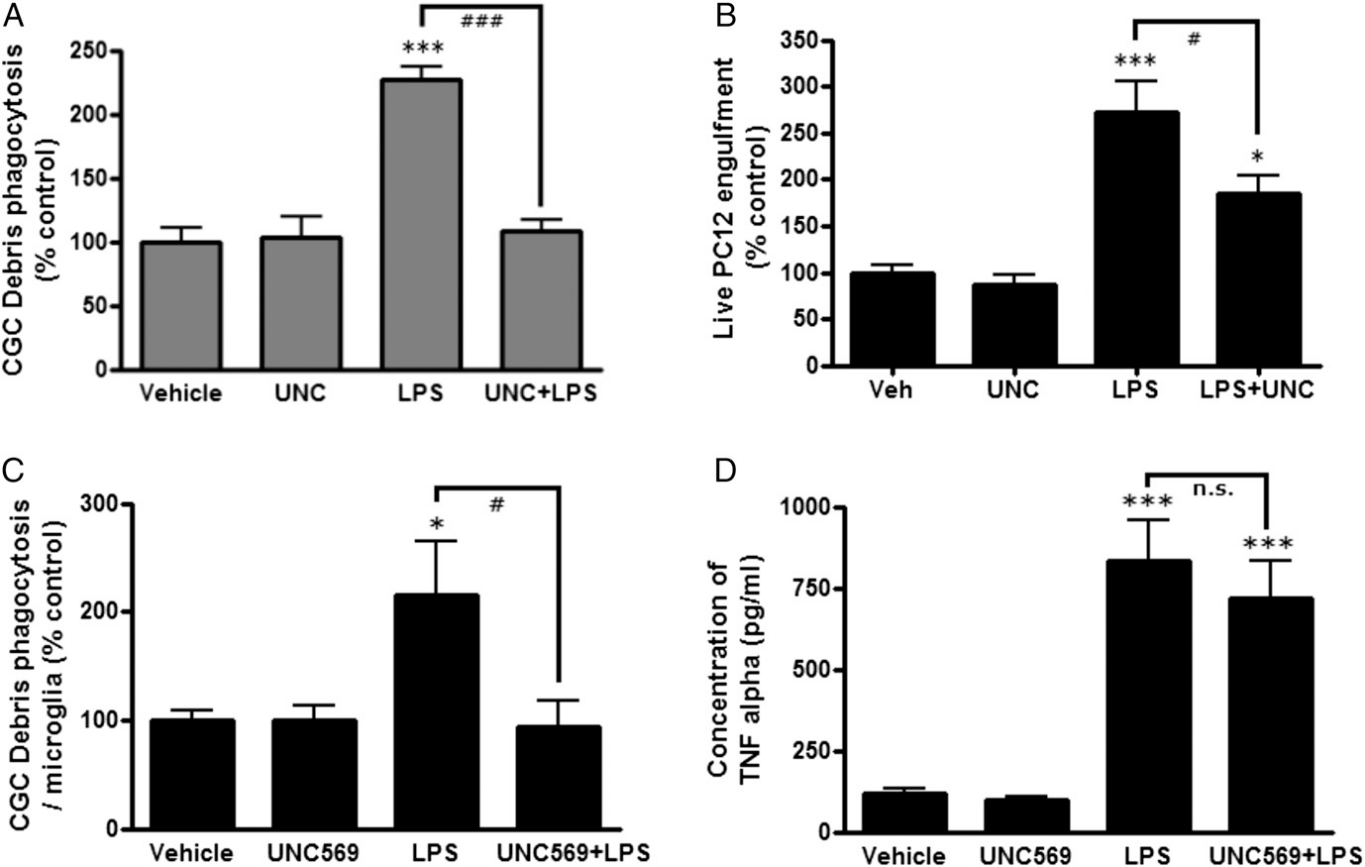
MerTK inhibitor UNC569 blocks LPS-induced phagocytosis. (**A**) BV-2 cells were treated or not with UNC569 (500 nM) for 30 min and then were treated or not with 100 ng/ml LPS for 24 h. Neuronal debris was added for 2 h, and the amount of debris that was phagocytosed was quantified. Absolute phagocytosis of the vehicle control cells: 6.57 ± 2.47% (*n* = 3). (**B**) Alternatively, live PC12 cells were added for 4 h instead of debris, and the amount of BV-2 phagocytosis of PC12 cells was quantified. Absolute phagocytosis of the vehicle control cells: 10.68 ± 0.98% (*n* = 5). (**C**) Primary glial cultures were treated or not with UNC569 (500 nM) for 30 min and then were treated or not with 100 ng/ml LPS for 24 h, neuronal debris was added for 2 h, and the amount of debris that was phagocytosed was quantified. Absolute phagocytosis of the vehicle control cells: 11.92 ± 2.70% (*n* = 3). (**D**) TNF-α levels in culture media were measured in the same experiment (mean ± SEM; *n* = 4). *p < 0.05, ***p < 0.001 versus vehicle control in the absence of LPS. ^#^*p* < 0.05, ^###^*p* < 0.001 LPS versus LPS + UNC569. All *p* values were calculated using the Tukey post hoc test.

Gal-3 can opsonize cells for phagocytosis via the phagocytic receptor MerTK (11). To test whether the Gal-3–induced phagocytosis of PC12 cells was mediated by MerTK, we added a MerTK-specific inhibitor, UNC569 (500 nM) (36), and found that this completely prevented the Gal-3–induced phagocytosis of neuronal debris (*p* < 0.001) and live PC12 cells (*p* < 0.001, Tukey post hoc test) (Fig. 3A, 3B). At this concentration, UNC569 had no effect on microglial viability or proliferation (Supplemental Fig. 1D). UNC569 also inhibited LPS-induced microglial phagocytosis of live PC12 cells (*p* = 0.033, Tukey post hoc test) (Fig. 4B, Supplemental Fig. 3C). LPS activation of the microglia increased their phagocytosis of neuronal debris, and this increase was prevented by blocking MerTK with UNC569 (*p* < 0.001) (Fig. 4A). In primary cultures of rat cortical glia, microglial phagocytosis of neuronal debris was significantly increased by LPS to 216.1 ± 48.2% (*p* = 0.039, Tukey post hoc test), and this increase was completely blocked by UNC569 (*p* = 0.028, Tukey post hoc test) (Fig. 4C). In these same primary cultures, LPS greatly increased TNF-α secretion (*p* < 0.001, Tukey post hoc test), but UNC569 did not inhibit this (*p* = 0.758, Tukey post hoc test) (Fig. 4D), indicating that UNC569 does not block LPS-induced phagocytosis by inhibiting the inflammatory response.

**FIGURE 5.**
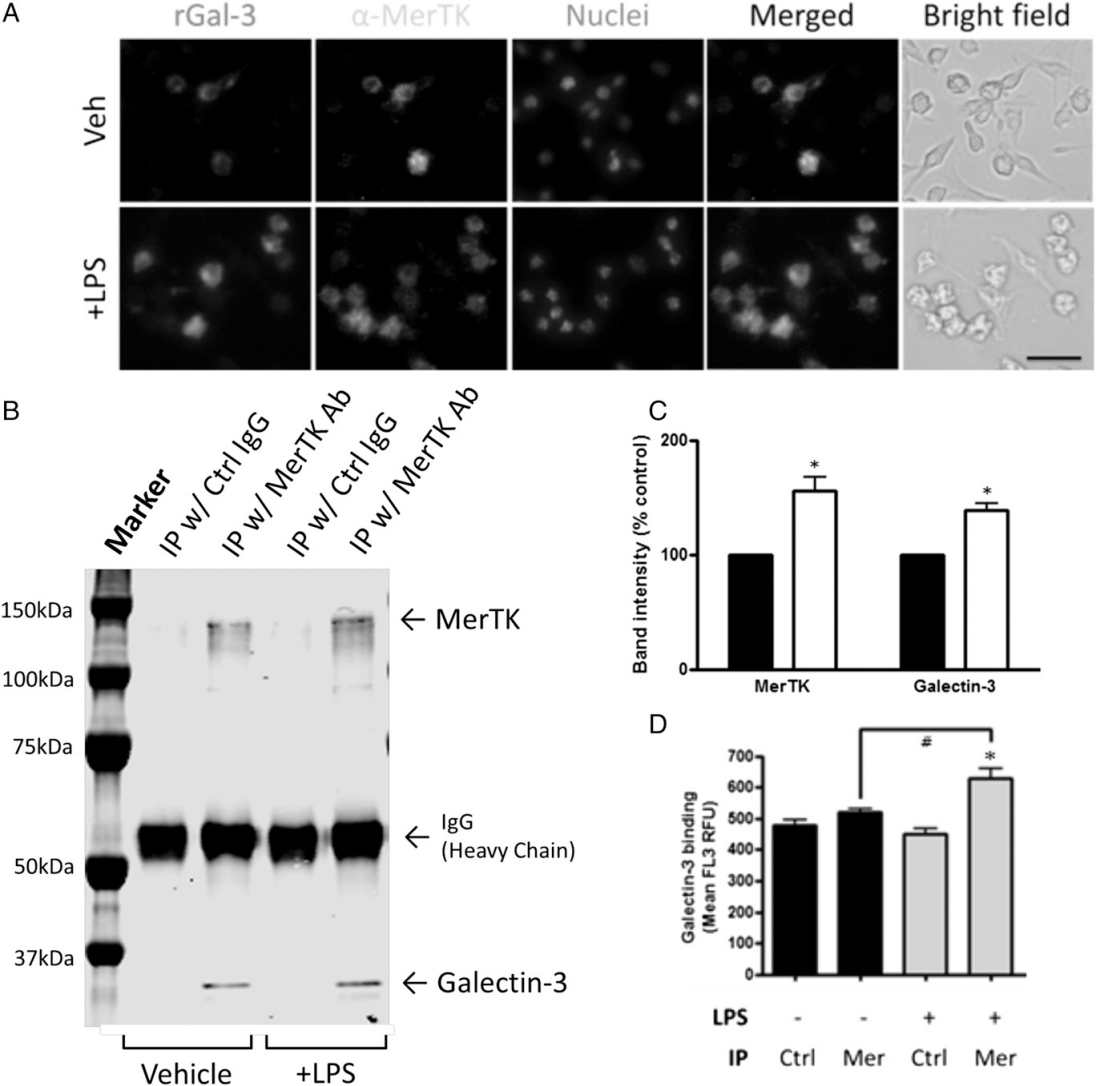
LPS upregulates MerTK expression and Gal-3 binding to BV-2 cells. (**A**) MerTK expression and Gal-3 binding were increased in response to LPS stimulation. Note that Gal-3 can bind cells that express MerTK. Scale bar, 50 μm. (**B**) Representative coimmunoprecipitation. A total of 250 μg of cellular lysates was immunoprecipitated with MerTK Ab or control IgG, and then Western blotted with MerTK and Gal-3 Abs. (**C**) Quantification of coimmunoprecipitation. MerTK and Gal-3 physically interact, and this interaction was significantly increased by LPS stimulation. Black bars, vehicle treatment; white bars, LPS treatment. *p < 0.05 versus vehicle (absence of LPS, immunoprecipitated with MerTK Ab). *n* = 3. (**D**) Gal-3 binding to immunoprecipitated MerTK. Cellular lysates were immunoprecipitated with MerTK Ab (C terminus) or control IgG and then immunoprecipitated beads were incubated with TAMRA-stained rGal-3 at 37°C for 1 h. Mean FL3 of beads were measured. *p < 0.05 versus corresponding control IgG group. ^#^*p* < 0.05 versus vehicle (absence of LPS, immunoprecipitated with MerTK Ab). *n* = 3 independent experiments each.

To determine whether Gal-3 colocalizes with MerTK on microglia, the binding of fluorescently labeled Gal-3 was compared with the binding Abs to MerTK on control and LPS-activated microglia. LPS increased Gal-3 binding and MerTK expression, and Gal-3 bound predominantly to MerTK-expressing cells (Fig. 5A). To test whether endogenous Gal-3 directly bound to MerTK, MerTK was immunoprecipitated from control and LPS-activated microglia, and the immunoprecipitate was subjected to Western blot for MerTK and Gal-3. Endogenous Gal-3 was clearly present in the MerTK immunoprecipitate but not that of a control Ab, and LPS increased the amounts of MerTK and Gal-3 in the immunoprecipitate (Fig. 5B, 5C). To test whether exogenous Gal-3 could bind to MerTK, MerTK was immunoprecipitated as before from control and LPS-activated microglia using an anti-MerTK Ab bound to beads, and the binding of fluorescent Gal-3 to these beads was quantified by flow cytometry. Gal-3 bound to the MerTK immunoprecipitate but not to that of a control Ab, and the MerTK binding was increased by LPS activation of the cells (*p* = 0.032 versus vehicle treatment with MerTK immunoprecipitated, Tukey post hoc test) (Fig. 5D).

To test whether Gal-3 bound directly to MerTK, rather than through some intermediate, we assayed whether purified Gal-3 bound to purified MerTK extracellular domain by fluorescence polarization. The fluorescence polarization of fluorescently labeled Gal-3 was substantially enhanced by the addition of increasing concentrations of MerTK ectodomain, indicating direct binding (Supplemental Fig. 4). This fluorescence enhancement was half saturated at 155 nM MerTK, indicating a *K*_d_ = 155 nM, assuming a 1:1 binding. As positive control, we used modified citrus pectin, which is known to bind Gal-3 with high affinity; we found that this enhanced the fluorescence polarization of Gal-3 to a similar extent as MerTK ectodomain. As negative control, we used the purified ectodomain of the microglial receptor TREM2 and found that this had no significant binding to Gal-3 (Supplemental Fig. 4).

Taken together, these findings suggest that Gal-3 binds to MerTK on microglia and can opsonize cells for microglial phagocytosis via MerTK.

### Neuraminidase

**FIGURE 6.**
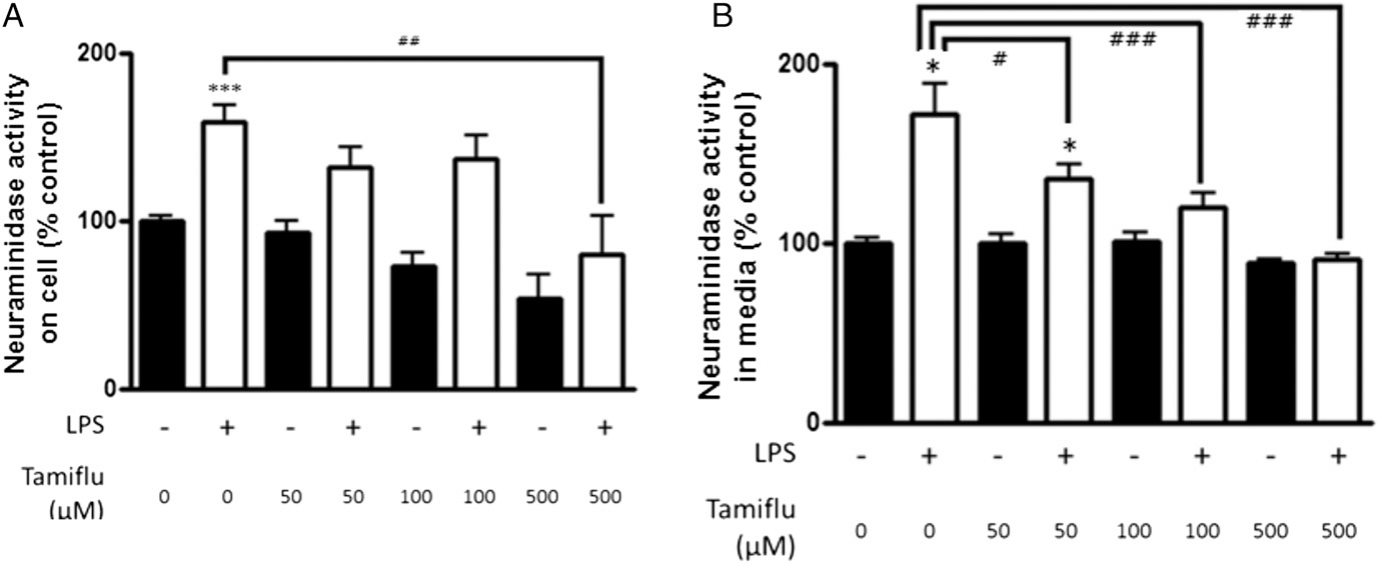
Neu in microglia. (**A** and **B**) BV-2 cells were exposed to vehicle or 100 ng/ml LPS for 24 h, following pretreatment with different concentrations of Tamiflu or vehicle. Neu activity was increased by LPS on the cellular surface (A) and in medium (B). *n* = 3 independent experiments for each; measurements were made in triplicate for each experiment. *p < 0.05, ***p < 0.001 versus untreated control. ^#^*p* < 0.05, ^##^*p* < 0.01, ^###^*p* < 0.001 versus LPS-treated control.

LPS has been found to upregulate Neu1 on the surface of macrophages, and the activity of this Neu has been found to be inhibited by Tamiflu (20). We found that LPS activation of microglia significantly increased Neu activity on the cellular surface (158 ± 11%) and in culture medium (171 ± 17%) (*p* < 0.001, *p* = 0.037, respectively, Tukey post hoc test) (Fig. 6), and the LPS-induced Neu activity on the cells and in the medium was completely inhibited by 500 μM Tamiflu (Fig. 6).

**FIGURE 7.**
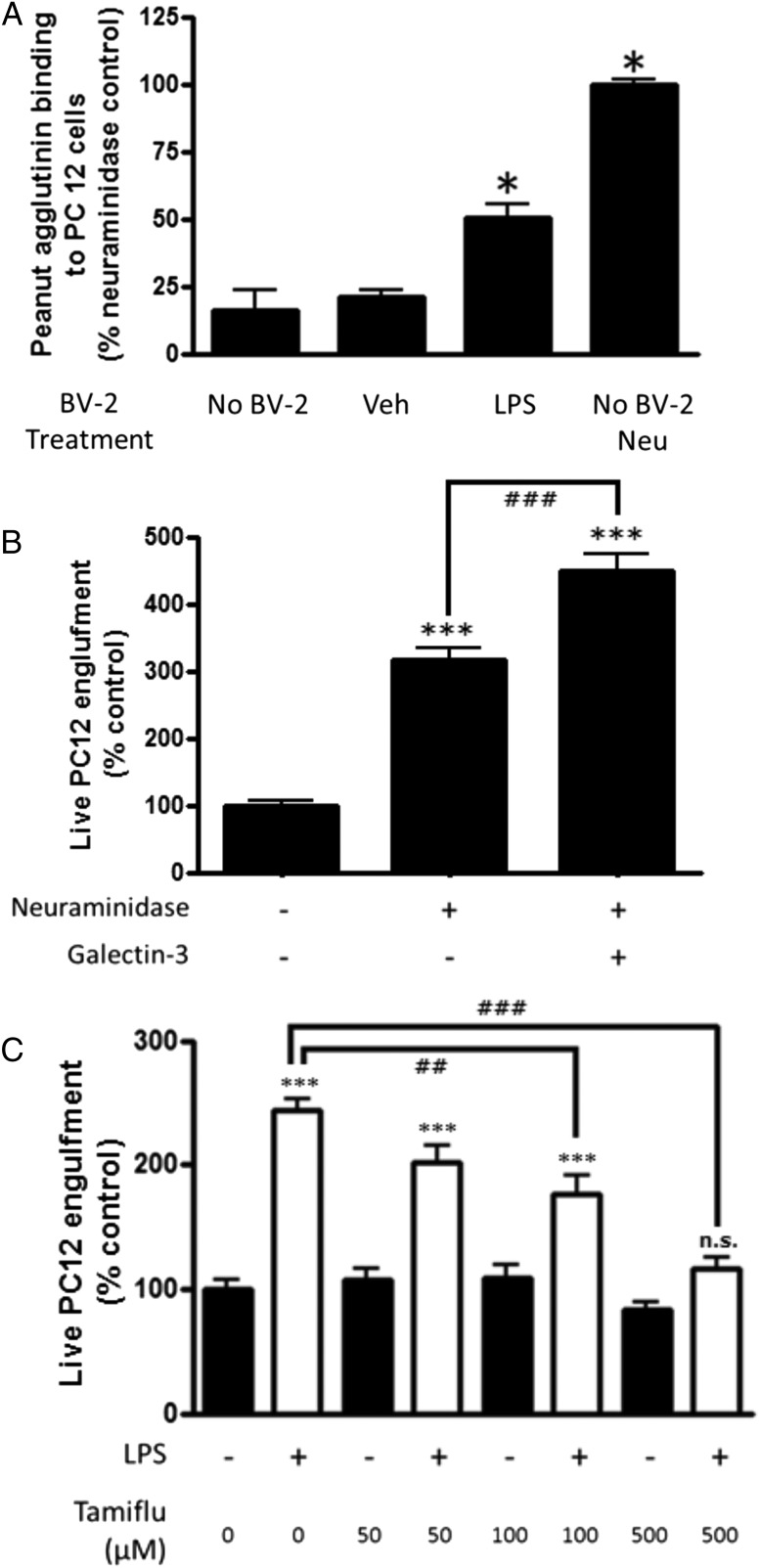
Activated microglia exhibit a Tamiflu-inhibitable Neu activity that enhances phagocytosis of PC12 cells. (**A**) Fresh culture medium (Veh) or culture medium incubated with BV-2 with or without LPS for 24 h was incubated with PC12 cells for 2 h (or alternatively, 0.1 U/ml Neu was added), and binding of peanut agglutinin was measured by flow cytometry. (**B**) Neu enhances microglial phagocytosis of live PC12 cells. BV-2 cells were exposed to vehicle or 0.1 U/ml Neu, with or without 200 nM Gal-3, for 1 h. Absolute phagocytosis of the vehicle control cells: 12.50 ± 1.19%. (**C**) LPS-induced microglial phagocytosis of live PC12 cells is inhibited by Tamiflu. Absolute phagocytosis of the vehicle control cells: 11.61 ± 1.07%. *n* = 3 for phagocytosis assay, and *n* = 4 for Neu activity assay. *p < 0.05 versus untreated PC12 cells, ***p < 0.001 versus untreated control. ^##^*p* < 0.01, ^###^*p* < 0.001 between the indicated treatments. All *p* values were calculated using the Tukey post hoc test. n.s., not significant.

We tested whether the sialidase activity exhibited by activated microglia was sufficient to desialylate PC12 cells. Peanut agglutinin binding to PC12 cells was unaffected by incubation with medium from nonactivated microglia, but it was increased 3-fold by incubation with medium from LPS-activated microglia; for comparison, it was increased 6-fold by incubation with 0.1 U/ml Neu (Fig. 7A). This indicates that the Neu activity secreted by activated microglia was sufficient to desialylate PC12 cells and that the level of desialylation is comparable to that induced by added Neu.

Because activation of microglia with LPS caused a Neu activity that desialylated PC12 cells, we tested whether Neu alone was sufficient to induce microglial phagocytosis of PC12 cells. Addition of extracellular Neu increased microglial phagocytosis of live PC12 cells to 316 ± 18% (*p* < 0.001, Tukey post hoc test) (Fig. 7B). Note that addition of Neu to PC12 cells alone had absolutely no effect on their viability (Supplemental Fig. 3C). Addition of Gal-3 together with Neu increased microglial phagocytosis of the PC12 cells to 449 ± 24% (*p* < 0.001 versus Neu alone, Tukey post hoc test) (Fig. 7B). Furthermore, LPS-induced phagocytosis of live PC12 cells was inhibited by the Neu inhibitor Tamiflu (*p* = 0.001 at 100 μM, *p* < 0.001 at 500 μM, Tukey post hoc test) (Fig. 7C). Thus, activated microglia exhibit Neu activity, inhibition of this Neu activity prevents phagocytosis of live PC12 cells by activated microglia, and Neu alone is sufficient to induce phagocytosis, which is enhanced by added Gal-3.

## Discussion

Gal-3 has been described as a ligand for MerTK on macrophages (11). Consistent with this, we found that MerTK and Gal-3 colocalized, Gal-3 induced microglial engulfment of cells, and this engulfment was blocked by the MerTK inhibitor UNC569. Gal-3 was rapidly released from LPS-activated microglia and bound to microglial cells and PC12 cells (especially to stressed or dying PC12 cells), as well as to MerTK from microglia. These and previous findings (11) suggest that Gal-3 can opsonize cells for phagocytosis by binding MerTK on phagocytes and some factors on target cells. Gal-3 binding to cells was blocked by lactose, as was LPS-induced phagocytosis of PC12 cells. Thus, Gal-3 appears to bind phagocyte and target cells via binding sugars, and this binding appears to be required for phagocytosis. Gal-3 knockdown prevented LPS-induced microglial engulfment of live PC12 cells. Thus, Gal-3 appears to act as an opsonin mediating the phagocytosis of cells by activated microglia.

What does Gal-3 bind to on target cells? Our results indicate that Gal-3 binds to desialylated sugar chains, as indicated by inhibition of cellular binding by lactose, which greatly increased binding after desialylation of the cells, and colocalization of binding with peanut agglutinin. We cannot be sure that Gal-3 did not enter the cells over the time course of the binding experiments; however, this seems unlikely because lactose completely prevented association with the cells. Thus, Gal-3 may act as an opsonin for desialylated cells or parts of cells that are desialylated.

LPS-activated microglia released a Neu activity into the medium, and this Neu activity was sufficient to desialylate PC12 cells. Neu1 may be responsible for this Neu activity, because it was inhibited by Tamiflu. This is consistent with reports that LPS and other inflammatory stimuli cause translocation of Neu1 to the cell surface of macrophages and other leukocytes, where it can desialylate cell surface glycoproteins (20, 21, 37). Added Neu and Gal-3 greatly increased microglial phagocytosis of PC12 cells. LPS-induced phagocytosis of PC12 cells was prevented by Tamiflu, lactose, knockdown of Gal-3, or inhibition of MerTK. This is consistent with LPS inducing microglial release of Neu that desialylates PC12 cells, allowing Gal-3 to bind and opsonize PC12 cells also via binding and activating MerTK on the microglia.

Gal-3 is certainly not the only opsonin, and MerTK is not the only receptor, that mediates microglial phagocytosis of neurons. Our findings that lactose inhibition of Gal-3 binding, Gal-3 knockdown, and UNC569 inhibition of MerTK do not fully inhibit LPS-induced phagocytosis of cells (Figs. 3C, 3E, 4B) are consistent with other opsonins and receptors being involved. Other opsonins that are known to mediate phagocytosis via MerTK are growth arrest–specific protein 6 and Protein S, which bind to exposed phosphatidylserine, whereas vitronectin receptors bind to exposed phosphatidylserine via the opsonin MFG-E8, the LRP1 receptor binds to calreticulin exposed on cells, and the CR3 receptor binds to iC3b-opsonized and, possibly, C1q-opsonized cells (2). The point is not that Gal-3 is the only opsonin but rather that it is a novel opsonin that binds a novel eat-me signal (i.e., desialylated cells). It is possible that Gal-3 and MerTK are both required for phagocytosis but do not directly interact to mediate phagocytosis. However, the finding in this study that they directly interact suggests that their coinvolvement is due to their direct interaction as opsonin and receptor.

In principle, Gal-3 could mediate phagocytosis by binding other phagocytic receptors, as well as MerTK. Because the binding of Gal-3 to microglia was prevented by lactose, it is possible that the binding of Gal-3 to MerTK (and potentially other receptors) was mediated by the carbohydrate recognition domain of Gal-3 binding to desialylated MerTK (and potentially other receptors). However, MerTK appears to be the main receptor by which Gal-3 increases phagocytosis, because a MerTK inhibitor prevented Gal-3–induced phagocytosis (Fig. 3A, 3B). We did not test directly whether MerTK mediated the phagocytosis of desialylated cells, but this seems likely given that MerTK mediated Gal-3–induced phagocytosis, and Gal-3 bound to desialylated cells.

Gal-3 increases after neonatal brain ischemia, and Gal-3–knockout mice are protected from the subsequent neuronal loss (38). We found that MerTK mutant rats were protected from transient ischemia–induced delayed neuronal loss mediated by microglial phagocytosis (8). This may be consistent with delayed neuronal loss after brain ischemia being due to Gal-3–, Neu1-, and MerTK-mediated microglial phagocytosis. Brain ischemia upregulates the microglial expression of MerTK (8) and Gal-3 (39), and this 7may be mediated, in part, by TLR4, because TLR4 is highly expressed in microglia during ischemia (e.g., 40) and has multiple potential ligands, including HMGB1, that are upregulated by ischemia (41).

Gal-3 appears to mediate microglial phagocytosis of myelin, which may contribute to demyelination or myelin debris clearance (42, 43), and the results from this study suggest that this might be a result of the desialylation of the myelin. Activated microglia can phagocytose live glioma cells (44), and the mechanisms described in this article might contribute to protection against brain cancers. Microglia phagocytose activated neutrophils to protect the brain from excessive inflammation (45), and activated neutrophils are known to desialylate via Neu1 translocation (37), which might contribute to their removal via the mechanisms described in this article. We recently found that Gal-3 is upregulated in a mouse model of brain trauma and that Gal-3–knockout mice have less neuronal loss posttrauma, consistent with Gal-3 mediating this neuronal loss (46).

## Supplementary Material

Data Supplement
