# Supplementary material for: Activated Microglia Desialylate and Phagocytose Cells via Neuraminidase, Galectin-3, and Mer Tyrosine Kinase
Source: J Immunol. 2017 May 12;198(12):4792–801. doi: 10.4049/jimmunol.1502532 (PMC5458330; doi:10.4049/jimmunol.1502532)
Supplement: Data Supplement [file JI_1502532.zip › JI_1502532_Supplemental_Figures_1.pdf]

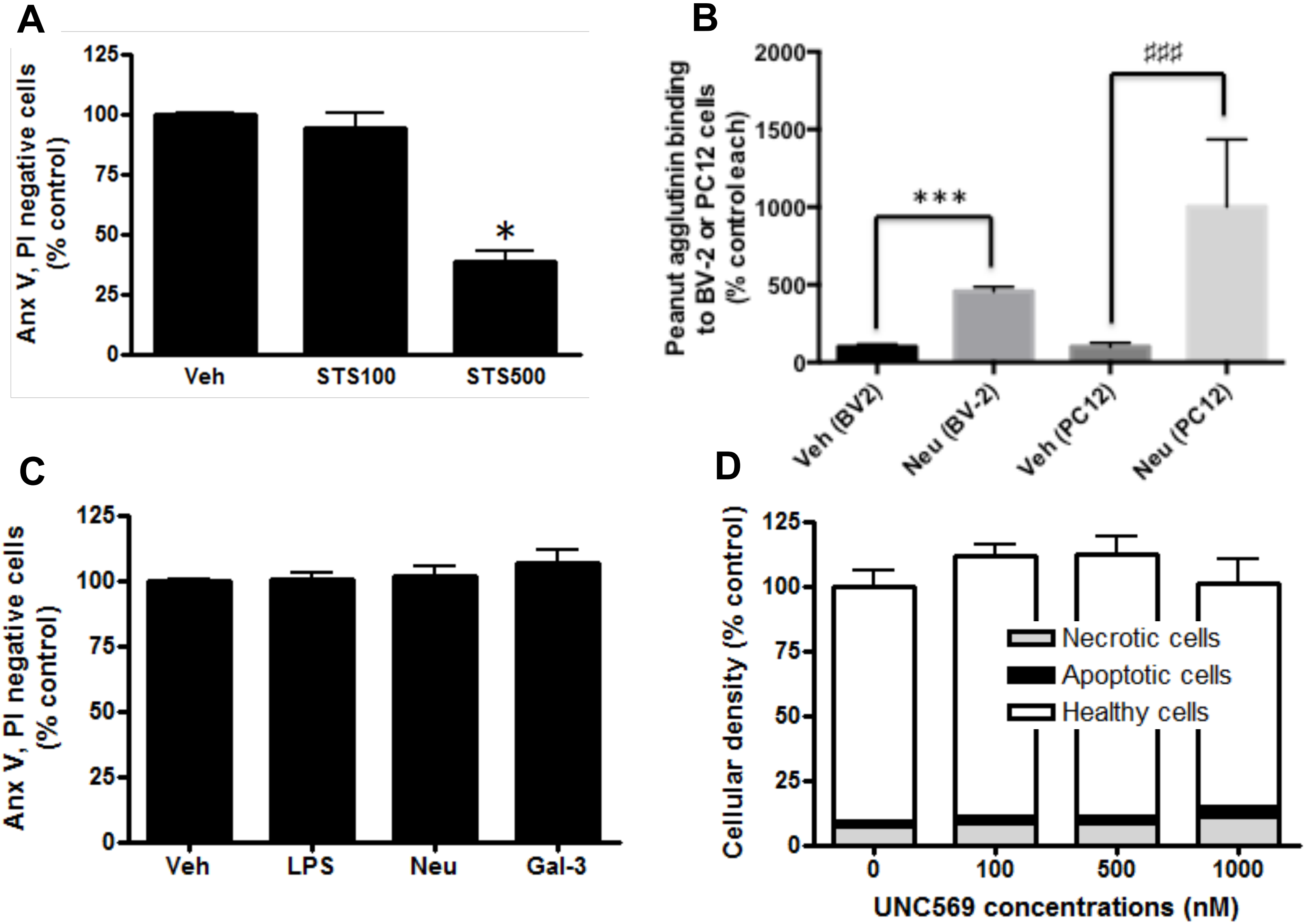

Supplementary Figure 1: ***A)*** Viable PC12 cells in the presence of 0, 100 and 500 nM staurosporine (STS) quantified by flow cytometry as cells not binding annexin V or propidium iodide. n=3. *p < 0.05 vs. no STS vehicle (Tukey's post-hoc test). ***B)*** Peanut agglutinin binding to BV-2 and PC12 cells ± neuraminidase (0.1U/ml). n=3. ***C)*** Viable PC12 cells in the presence of LPS (100ng/ml), neuraminidase (0.1U/ml) and Galectin-3 (200nM) quantified as cells not binding annexin V or propidium iodide. n=3. **D*)*** BV-2 density and viability in the presence of 0, 100, 500 and 1000 nM UNC569. n=3.

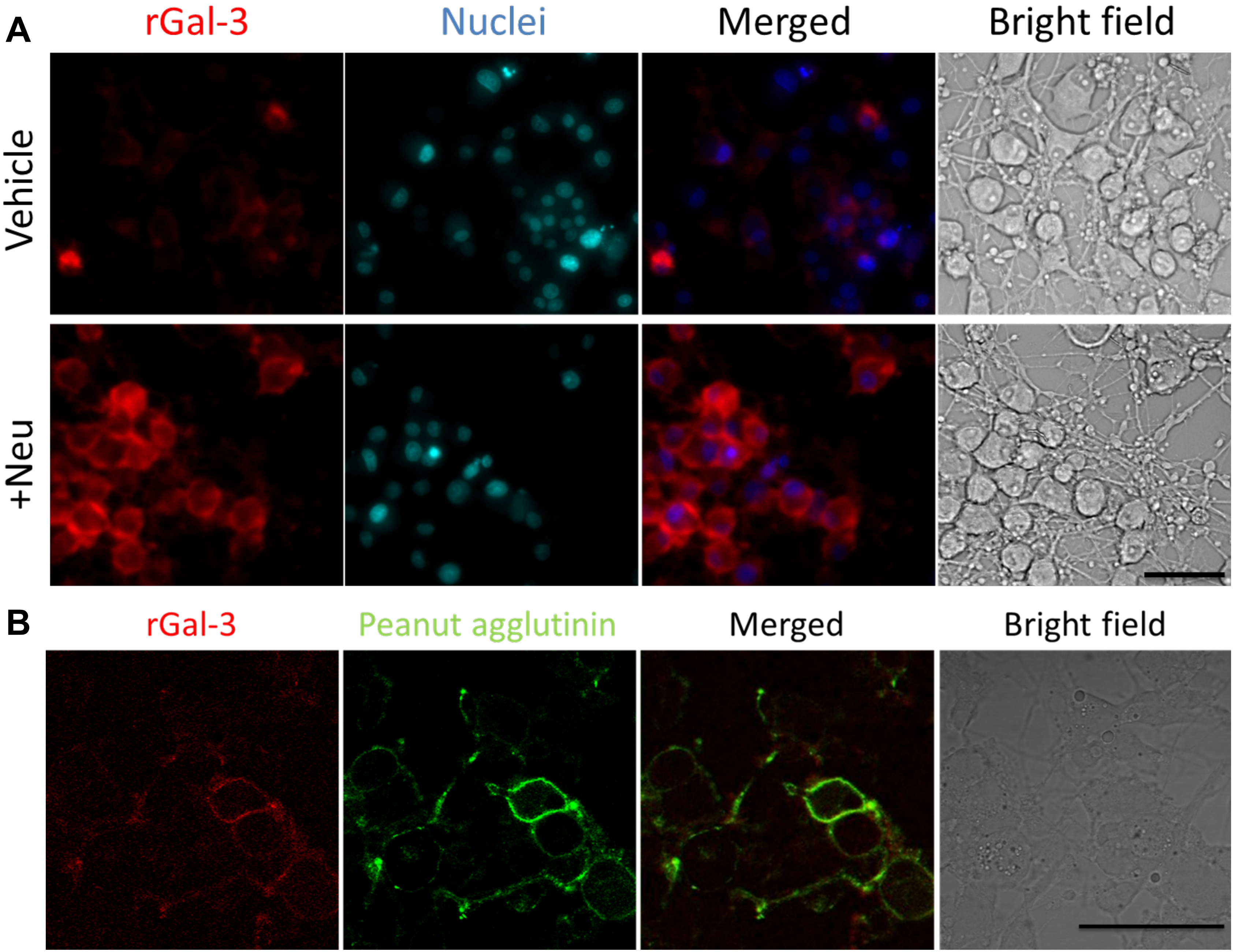

Supplementary Figure 2: Desialylation of PC12 cells enables galectin-3 to bind. ***A)*** Fluorescence microscope image of galectin-3 binding. Neuraminidase increases galectin-3 binding to PC12 cells. ***B)*** Confocal microscope image of galectin-3 and peanut agglutinin binding. Galectin-3 and peanut agglutinin are co-localised on the surface of PC12 cells. Scale bars = 50 µm. Representative images.

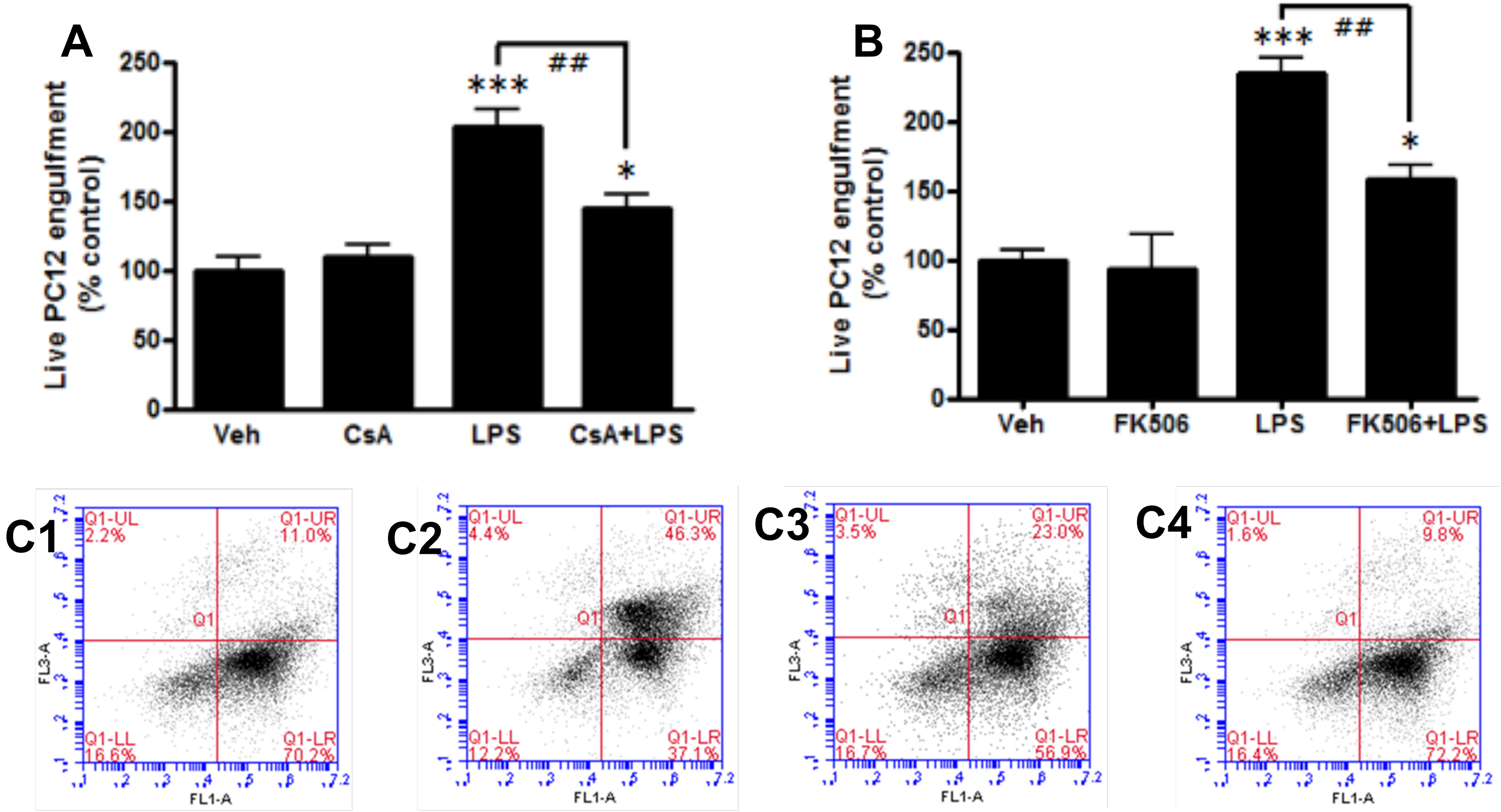

Supplementary Figure 3: LPS-induced phagocytosis of PC12 cells in blocked by calcineurin inhibitors or UNC569

BV-2 cells were exposed to vehicle or 100 ng/ml of LPS for 24 hours, following pre-treatment with vehicle (Veh) or A) Cyclosporine A (CsA), B) FK506 or C) UNC569. n=3. *$p < 0.05$ and ***$p < 0.001$ vs. no inhibitor group in the absence of LPS; ##$p < 0.01$ vs. corresponding vehicle (no inhibitor) treatment in the presence of LPS (Tukey's post-hoc test). C) Example of flow cytometry of phagocytosis of PC12 cells. ***c) i)*** Vehicle treatment ***ii)*** LPS treatment ***iii)*** UNC569 pre-treatment and LPS treatment ***iv)*** UNC 569 treatment only. Note that FL1 reflects BV-2 cells (stained with Alexa488-conjugated IB_4) and FL3 reflects PC12 cells (stained with TAMRA). Therefore, population in upper left: PC12 positive control, lower right: BV-2 negative control and upper right: target (phagocytosing), and changes in mean FL3 in upper right + lower right were analysed.

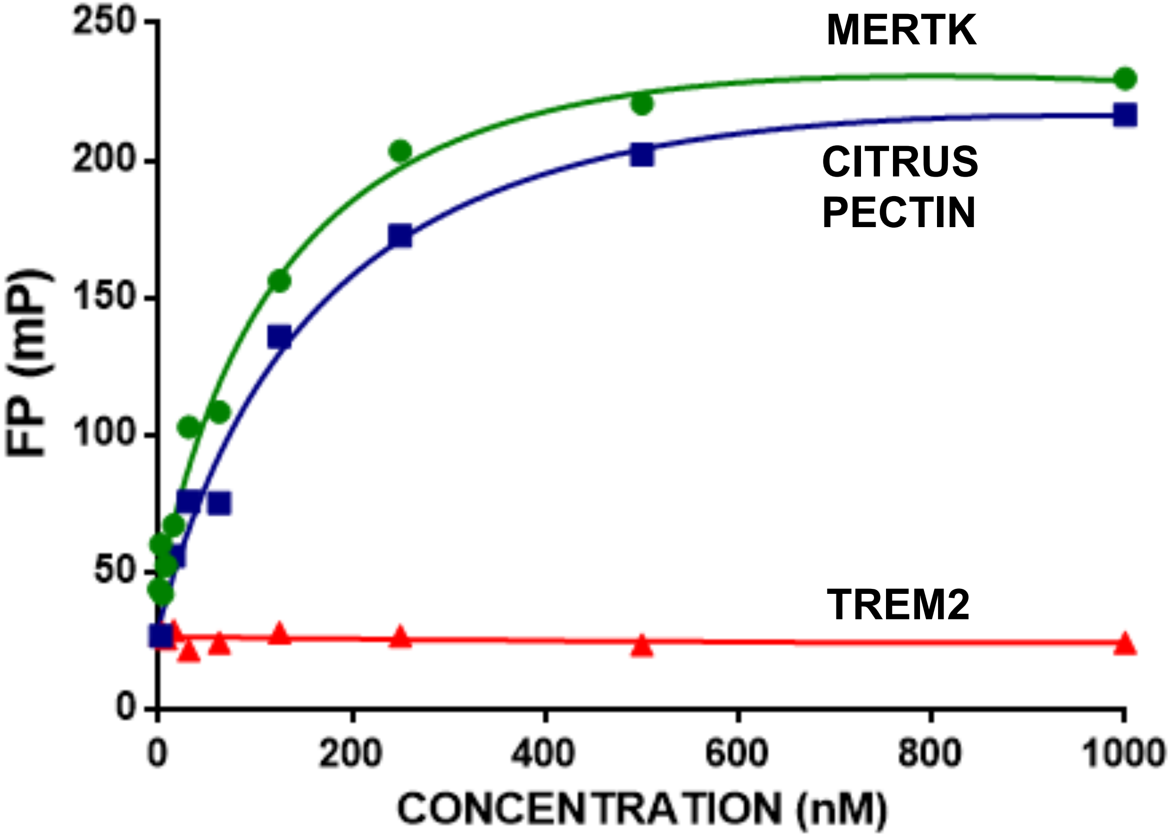

Supplementary Figure 4: Galectin 3 binds to MerTK. Fluorescence polarisation experiments for MerTK ectodomain, modified citrus pectin (positive control) and TREM2 ectodomain (negative control) showed that galectin-3 binds to MerTK and citrus pectin, but not to TREM2. The K_d estimated by GraphPad Prism for MerTK was 155nM. Modified citrus pectin has a range of molecular weight, but 1000 nM on the graph corresponds to 30 mg/ml, and the estimated K_d was 66 µg/ml. Representative result of two independent experiments.
